# Aluminium-Induced Oxidative Stress, Apoptosis and Alterations in Testicular Tissue and Sperm Quality in Wistar Rats: Ameliorative Effects of Curcumin 

**DOI:** 10.22074/ijfs.2017.4859

**Published:** 2017-09-03

**Authors:** Ebrahim Cheraghi, Alireza Golkar, Kambiz Roshanaei, Behrang Alani

**Affiliations:** 1 Department of Biology, Faculty of Sciences, University of Qom, Qom, Iran; 2 Sciences Research Laboratory, Department of Biology, Qom Branch, Islamic Azad University, Qom, Iran; 3 Department of Biology, Qom Branch, Islamic Azad University, Qom, Iran; 4 Department of Applied Cell Sciences, Faculty of Medicine, Kashan University of Medical Sciences, Kashan, Iran

**Keywords:** Aluminum, Toxicity, Curcumin, Male Reproductive System, Oxidative Stress

## Abstract

**Background:**

Reproductive toxicity is a major challenge associated with aluminum (Al) exposure. No studies have evaluated the possible effects of curcumin (CUR) on Al-induced reproductive dysfunction. Therefore, this study investigated the effects of CUR treatment on Al-induced reproductive damage.

**Materials and Methods:**

In this experimental study, 40 male Wistar rats were allocated to the five groups (n=8) based on the treatment they received: no treatment (control), solvent [dimethyl sulfoxide (DMSO) or distilled water], CUR 10 mg/kg body weight (BW), Al chloride 10 mg/kg BW, and CUR+Al chloride (10 mg/kg BW/each alone). Treatments were performed by intraperitoneal (IP) injections for 28 days. The left testis was assessed for histopathological analysis as well as the incidence of germ cell apoptosis. One-way analysis of variance (ANOVA) followed by the Tukey’s test was used. P<0.05 was considered significant. A value of P<0.05 was considered significant.

**Results:**

Significant reductions in body and testis weight; plasma testosterone and luteinizing hormone levels; sperm count, motility, morphology, and viability; germinal epithelium thickness; seminiferous tubules diameter; as well as, superoxide dismutase activity were observed in rats treated with Al. Moreover, Al exposure caused significant increments in the lumen diameter of tubules, terminal deoxynucleotidyl transferase dUTP nick end labeling (TUNEL)-positive cells and malondialdehyde (MDA) levels compared to the control group. However, in rats receiving CUR+Al, CUR significantly reversed the adverse effects of Al on testis and sperm quality. No significant differences in follicle-stimulating hormone (FSH) levels and nuclear diameter of spermatogonia were detected among all groups.

**Conclusion:**

It can be concluded that Al causes reproductive dysfunction by creating oxidative damage. CUR, on the other hand, reduces the toxic effects of Al and improves the antioxidant status and sperm quality in male rats.

## Introduction

Aluminum (Al) is the most common metallic element and the third most common element in the Earth’s crust (1). The ionic form of this metal is detectable not only in all natural waters, but also most types of animal and plant tissues. Due to its reactivity, Al is naturally found in combination with other elements to form compounds such as Al sulfate and chloride (2). Al compounds are extensively used in a wide range of products from household cookware and storage utensils to water purification agents, pharmaceuticals (such as antacids, vaccines, anti-diarrhea drugs, phosphate binders, and allergy immunotherapy injections), food additives, and even toothpastes (3).

The great abundance of Al increases the risk of exposure and related health issues in humans (4). High consumption of Al-containing products will increase the concentration of this metallic element in the consumers’ organs and damage their various tissues (including the testicular tissues of humans and animals). Moreover, high levels of Al in spermatozoa and seminal plasma of humans have been reported to reduce sperm viability and motility (5, 6). Krasovskiĭ et al. (7) have confirmed the gonadal toxicity of lead and Al chloride in guinea pigs and rats. Guo et al. (8) have attributed the oxidative damage and testicular toxicity caused by Al to the reduction in testis acetyl cholinesterase (AChE) activity. Chinoy et al. (9) have also found the 30-day consumption of sodium fluoride and Al chloride to cause structural changes in the testis, such as formation of giant cells. Testicular Al accumulation, necrosis of spermatocytes/spermatids, and a significant reduction in fertility were also observed in both male rats and mice (10, 11). Al may cause male reproductive toxicity through various mechanisms such as inducing oxidative stress, interfering with spermatogenesis and steroidogenesis, impairing cell signaling, disrupting the blood-testis barrier, and affecting the endocrine system (12).

In recent years, increasing attention has been paid to the application of nutritional antioxidants (such as herbal products) in diseases related to oxidative stress. The protective effects of herbal products have been attributed to their role as free radical scavengers and antioxidant defense regulators (13). Curcumin (Curcuma longa Lin, CUR), such as the active component of turmeric, can serve as an antioxidant and therapeutic agent without any side effects (14). As a free radical scavenger, CUR can largely inhibit the production of reactive oxygen species (ROS) both *in vitro* and in vivo. It also exhibits anti-carcinogenic, anti-inflammatory, and antibacterial properties (15), as well as acts a potent cancer chemopreventive agent (16) and tumor cell proliferation inhibitor (17).

Despite the reported antioxidant properties of CUR (18-21), its effects on apoptosis, oxidative stress and sperm quality in Al-treated rats have not been investigated. Therefore, the present study analyzed the protective effects of CUR on Al-induced damage to the reproductive system of male rats.

## Materials and Methods

CUR powder (C21H20O6, Merck & Co. Inc., Germany) was dissolved in dimethyl sulfoxide (DMSO). Al chloride (Merck & Co. Inc., Germany) was diluted with distilled water before administration.

### Experimental protocol

In this experimental study, a total of 40 male Wistar rats (240-260 g) were obtained from the animal house of Razi Institute (Iran). Rats were housed in individually ventilated cages on a 12-hour light/dark cycle, temperature of 24 ± 2ºC, with water and food ad libitum. The experimental protocol was approved by the Animal Ethics Committee in accordance with the Guide for the Care and Use of Laboratory Animals prepared by Qom University of Medical Sciences (Qom, Iran).

Over a two-week adaptation period, all rats were fed by a standard pellet diet and closely monitored to ensure normal growth and behavior. The rats were then weighed and randomly allocated to five groups of eight animals (two control groups and three experimental groups) to receive the following treatments for 28 days (22, 23): group I (control group): no injections. Group II (control group): intraperitoneal (IP) injections of only the solvent (distilled water or DMSO). Based on the solvents, we chose two control groups, distilled water and DMSO. Since there were no significant solvents, between the results of the control groups, we considered data from the distilled water group as the control group. Group III (experimental group): IP injections of CUR 10 mg/kg body weight (BW) (22) in 0.2 ml DMSO. Group IV (experimental group): IP injections of Al chloride 10 mg/kg BW (23) in 0.2 ml distilled water. Group V (experimental group): IP injections of CUR+Al chloride at the above-mentioned doses alone.

All groups were fed by a normal diet. After the treatment period, the rats were reweighed that was followed by being euthanized and dissected. Blood samples were collected into heparinized capillary tubes through cardiac puncture. In order to separate the plasma, the samples were poured into clean tubes and centrifuged at 1500 g for 20 minutes at 4°C. Testis and epididymis were detached from the adhering connective tissues, washed in cold physiological saline, and weighed accurately.

### Plasma hormone assay

Plasma was obtained and maintained at -20°C until enzyme-linked immunosorbent assay (ELISA) was performed. The concentrations of luteinizing hormone (LH), follicle-stimulating hormone (FSH) and testosterone were determined using ELISA kits (Elabscience Biotechnology Co., Ltd., Germany) according to the manufacturer's instructions. All measurements were carried out in duplicate. The intra- and inter- assay coefficients of variation were less than 10%.

### Assessment of lipid peroxidation

Thiobarbituric acid reactive substance (TBARS) levels were determined as a measure of plasma concentrations of malondialdehyde (MDA), the end product of lipid peroxidation (LPO) (24). MDA levels were reported as nmol/ml.

### Assessment of superoxide dismutase levels

Superoxide dismutase (SOD) activity in plasma was measured using a commercial assay kit (Cayman Chemical, USA) according to the manufacturer's instructions. This kit utilized a tetrazolium salt for the detection of superoxide radicals generated by xanthine oxidase and hypoxanthine. One unit of SOD was defined as the amount of enzyme needed to exhibit 50% dismutation of the superoxide radical. The SOD assay measured all three types of SOD (Cu/Zn, Mn, and Fe SOD) in U/ml (25).

### Sperm parameters

#### Assessment of sperm motility and count

The right cauda epididymis was incised and semen was pressed on a pre-warmed slide. Two drops of warm 2.9% sodium citrate were added to semen and mixed with a coverslip. The percentage of sperm motility was evaluated visually (magnification: ×40). Motility estimates were performed from three different fields in each sample. The mean of the three successive estimations was used as the final motility score. For sperm count, the left cauda epididymis was incised and the dripped semen was quickly sucked into a red blood pipette to the 0.5 mark. The collected semen was diluted with warm normal saline up to the 101 mark. Approximately 10 μL of the semen mixture was placed on a Neubauer chamber and viewed (magnification: ×40). The total numbers of sperm cells were counted and expressed as 106/ml (26).

#### Assessment of sperm viability and morphology

Eosin/nigrosin staining was used to determine sperm viability (percentage of live spermatozoa). A drop of semen (50 μL) with two drops of the stain (100 μL) was placed on a microscope slide. Thin smears were then prepared and observed under a light microscope (magnification: ×100). While viable sperms remained colorless, nonviable sperms appeared red. The stained and unstained sperm cells were counted. The mean values for each group were then recorded and used in percentage viability calculation. In order to determine the percentage of morphologically abnormal spermatozoa, eosin-nigrosin staining was performed, and the slides were viewed under a light microscope (magnification: ×100). A total of 200 sperm cells were examined on each slide, and the head, tail and total abnormality rates of spermatozoa were expressed as a percentage (27).

#### Histological analysis

In brief, an abdominal incision was made, while the testes were carefully dissected and fixed in 10% formal- saline. After paraffin embedding, the sections of 5 μm thickness were obtained using a rotary microtome, stained with Heidenhain’s Azan, and observed under a light microscope (magnification: ×200) (28).

### TUNEL method for analysis of apoptosis

The in-situ DNA fragmentation was visualized by terminal deoxynucleotidyl transferase dUTP nick end labeling (TUNEL) method. Briefly, dewaxed testis sections were predigested with 20 mg/ml proteinase K for 20 minutes and incubated in phosphate buffered saline (PBS) solution containing 3% H_2_O_2_ for 10 minutes to block the endogenous peroxidase activity. The sections were incubated with the TUNEL reaction mixture, fluorescein-d UTP (Roche Applied Science, Germany), for 60 minutes at 37°C, according to the manufacturer’s instructions. The slides were then rinsed three times with PBS and incubated with secondary anti-fluorescein- POD-conjugate for 30 minutes. After washing three times in PBS, Hoechst stain (Sigma-Aldrich, USA) was added for chromogenic reaction. As a control for method specificity, the step using the TUNEL reaction mixture was omitted in negative control serial sections, so nucleotide mixture in reaction buffer was used instead. The apoptotic index was determined at 10-random locations within each seminiferous tubule. In all groups, 100 seminiferous tubules for each animal were recorded (29).

### Statistical analysis

The normality of continuous variables was confirmed using the Kolmogorov-Smirnov test. Data were reported as mean ± SE and analyzed with oneway analysis of variance (ANOVA) and Tukey’s test for post-hoc analysis. P<0.05 were considered significant. All analyses were performed with the Statistical Package for the Social (SPSS) for Windows 16.0 (SPSS Inc., USA).

## Results

### Effects of curcumin and aluminum on sperm characteristics, the testis and body weight

Significant reductions in sperm count (P=0.0001), motility (P=0.0001), viability (P=0.001), and morphology (P=0.001) were detected in rats treated with Al chloride compared to control group. Moreover, sperm parameters were significantly higher in rats treated with CUR alone (P=0.0001) compared to those treated with Al chloride, but this value was the same in control group (P>0.05). Rats treated with CUR+Al chloride had significantly higher sperm count (P=0.001), motility (P=0.006), viability (P=0.001), and morphology (P=0.001) compared to Al-treated rats (Table 1, Figes.1, 2). As Table 1 shows, while the testis and body weights were significantly reduced in Altreated rats (P=0.001) compared to control group, the values were similar in other groups (P>0.05).

**Fig.1:**
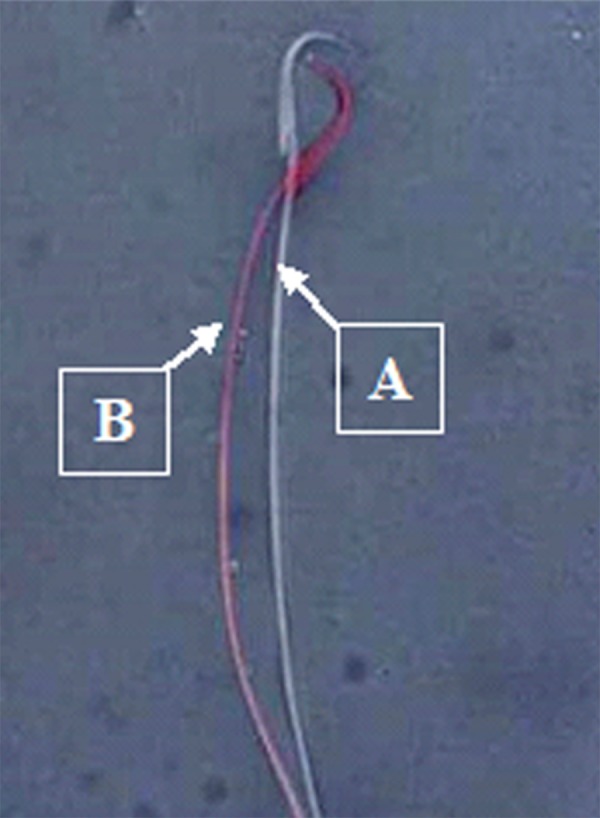
Assessment of sperm viability in rats treated by eosin-nigrosin stain (magnification: ×1000). **A.** Alive sperm and **B.** Dead sperm.

**Table 1:** Changes in body weight, testis weight, sperm count, sperm motility, sperm viability and sperm abnormalities in experimental groups


Parameter	Experimental group				
	Control	Solvent	CUR	Al	CUR+Al

Body weight (g)	276.1 ± 3.1^a^	279.6 ± 3.8^a^	284.4 ± 5.1^a^	245.2 ± 4.2^b^	280.6 ± 5.4^a^
Testis weight (g)	1.36 ± 0.04^a^	1.35 ± 0.05^a^	1.37 ± 0.03^a^	1.09 ± 0.02^b^	1.34 ± 0.04^a^
Sperm count (10^6^/ml)	36.22 ± 2.8^ac^	36.02 ± 3.5a^c^	45.4 ± 3.5^a^	17.5 ± 1.1^b^	32.6 ± 1.9^c^
Sperm motility (%)	84.8 ± 1.7^ac^	82.3 ± 3.1a^c^	87.1 ± 1.7^a^	43.9 ± 3.8^b^	68.3 ± 6.3^c^
Sperm abnormalities (%)	12.34 ± 1.5^ac^	12.5 ± 1.4a^c^	9.5 ± 0.7^a^	40.1 ± 3.2^b^	20.4 ± 2.8^c^
Sperm viability (%)	79.8 ± 2.4^a^	78.5 ± 2.7^a^	88.9 ± 1.9^a^	36.3 ± 3.2^b^	60.7 ± 2.7^c^


Data are shown as mean ± SE. Means within the same row with different letters are significantly differed (P<0.05) using ANOVA, Tukey's test. CUR; Curcumin and Al; Aluminum.

### Effect of curcumin and aluminum on reproductive hormones

We observed that testosterone and LH levels were significantly lower in Al-treated rats compared to other groups (P=0.01). Testosterone and LH levels were higher in CUR-treated group than all other groups. LH and testosterone levels in CUR+Al treated group were significantly (P=0.04) different from Al-treated group, but this value was not significantly different compared to control and CUR-treated rats (P>0.05, Table 2). There was also no significant differences in FSH levels among the five groups (P>0.05).

### Effects of curcumin and aluminum on lipid peroxidation status

Rats treated with Al chloride in comparison with other groups showed significantly increased MDA levels (P=0.01) and significantly decreased SOD activity (P=0.0001). Treatment with CUR+Al chloride resulted in significant improvement of LPO status when compared with Al-treated rats (P=0.001), but this value was not significantly different compared to control and CUR-treated rats (P>0.05, Table 2).

### Effects of curcumin and aluminum on the structure of the testes

Al chloride treatment led to degeneration and necrosis with a significant reduction in the diameter of seminiferous tubules and germinal epithelium thickness compared to the control group (P=0.0001). Moreover, the lumen diameter of tubules was significantly higher in Al-treated rats than in the control group (P=0.0001). This effect was milder in rats treated with CUR+Al chloride, while the values in this group were close to those in the control group. The diameter of seminiferous tubules, germinal epithelium thickness and the lumen diameter of tubules were similar in CUR-treated rats and control group (P>0.05, Table 3,Fig .3). In addition, we observed no significant difference in the nuclear diameter of spermatogonia among the groups (P>0.05, Table 3).

**Table 2:** The changes of FSH, LH, Testosterone, MDA and SOD levels in experimental groups


Parameter	Experimental group				
	Control	Solvent	CUR	Al	CUR+Al

FSH (IU/L)	2.33 ± 0.4^a^	2.35 ± 0.3^a^	2.3 ± 0.4^a^	2.7 ± 0.6^a^	2.13 ± 0.2^a^
LH (IU/L)	2.3 ± 0.42^a^	2.6 ± 0.4^a^	2.8 ± 0.45^a^	0.73 ± 0.07^b^	2.2 ± 0.3^a^
T (ng/ml)	3.7 ± 0.6^a^	3.6 ± 0.7^a^	4.1 ± 0.5^a^	1.3 ± 0.3^b^	3.6 ± 0.7^a^
MDA (nmol/ml)	4.8 ± 0.44^a^	5.01 ± 0.43^a^	4.1 ± 0.53^a^	7.4 ± 0.65^b^	5.16 ± 0.54^a^
SOD (U/ml)	8.26 ± 0.4^a^	8.27 ± 0.33^a^	8.93 ± 0.43^a^	3.26 ± 0.42^b^	7.5 ± 0.43^a^


Data are shown as mean ± SE. Means within the same row with different letters are significantly differed (P<0.05) using ANOVA, Tukey’s test. CUR; Curcumin, Al; Aluminum, FSH; Follicle stimulating hormone, LH; Luteinizing hormone, T; Testosterone, MDA; Malondialdehyde, and SOD; Superoxide dismutase.

**Table 3:** The changes of histopathology on rat testis in the experimental groups


Parameter	Experimental group				
	Control	Solvent	CUR	Al	CUR+Al

The diameter of seminiferous tubules (µ)	181.27 ± 0.8^a^	181.77 ± 0.6^a^	184.32 ± 1.5^a^	157.19 ± 1.2^b^	180.66 ± 1.3^a^
The lumen diameter of tubules (µ)	77.02 ± 1.5^a^	77.03 ± 1.4^a^	74.48 ± 1.45^a^	98.93 ± 0.73^b^	78.74 ± 0.82^a^
The nuclear diameter of spermatogonia (µ)	4.71 ± 0.01^a^	4.70 ± 0.01^a^	4.75 ± 0.02^a^	4.69 ± 0.01^a^	4.70 ± 0.01^a^
Germinal epithelium thickness (µ)	56.29 ± 0.4^a^	55.16 ± 0.8^a^	57.39 ± 0.6^a^	36.46 ± 0.6^b^	51.56 ± 0.9^a^


Data are shown as mean ± SE. Means within the same row with different letters are significantly differed (P<0.05) using ANOVA, Tukey’s test. CUR; Curcumin and Al; Aluminum.

**Fig.2:**
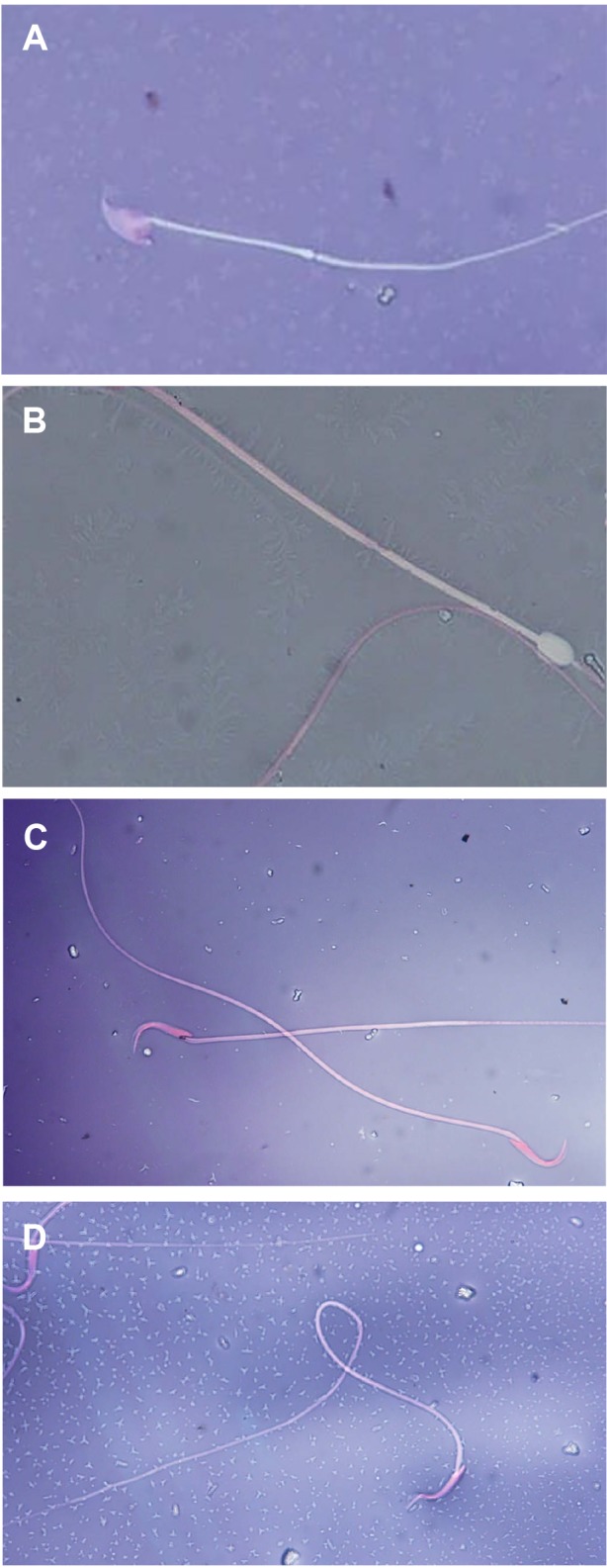
Some of abnormal sperm tail and head morphology after aluminum exposure by eosin-nigrosin stain (magnification: ×1000). **A.** Amorphous head, **B.** Cytoplasmic droplet, **C.** Amorphous mid-piece and tail, and D. Coiled or curled tail.

**Fig.3:**
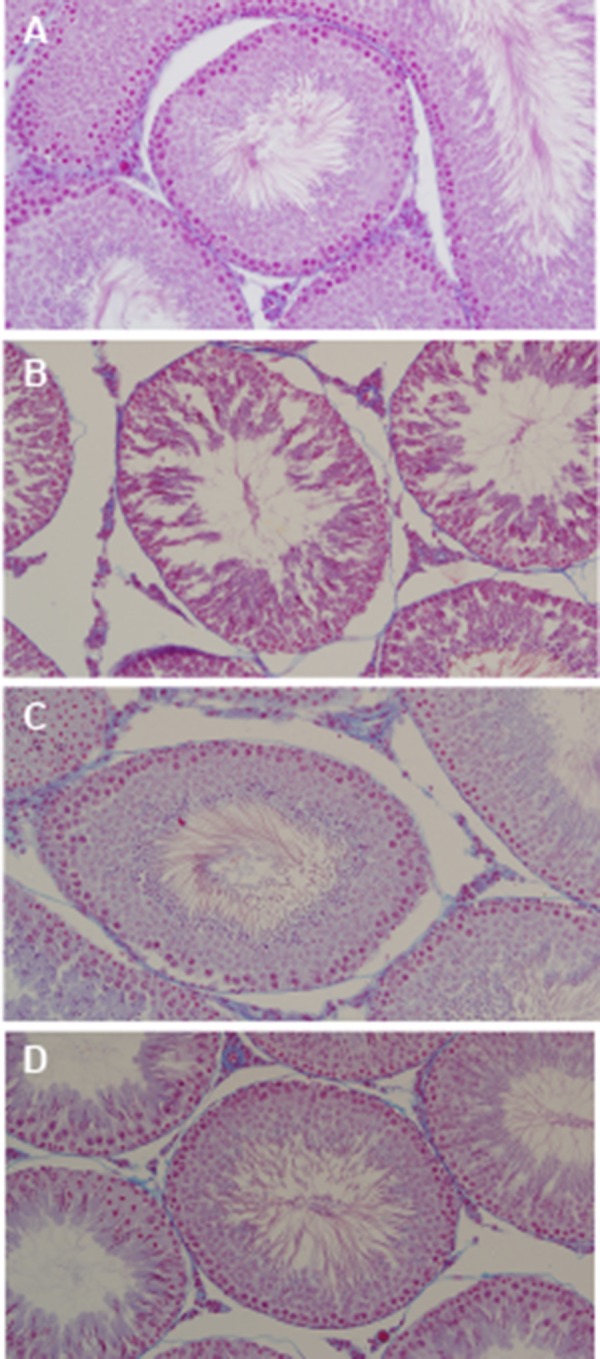
Photomicrographs of transverse sections in the testis (Heiden hain’s azan stain, 5 μm sections, magnification: ×100). **A.** Testis of control group showing normal histological structure of seminiferous tubules, **B.** Testis of aluminum group showing an increase in the lumen diameter of tubules, a decrease in the diameter of seminiferous tubules as well as distorted seminiferous tubules with loss of normal distribution of epithelial lining and vacuolar cytoplasm (black arrow), **C.**Testis of curcumin group showing no histological changes, and **D.** Testis of CUR+Al group revealed no histopathological changes.

### Effects of curcumin and aluminum on apoptosis

Rats treated with Al chloride showed significantly increased TUNEL-positive cells (P=0.001) in comparison with other groups. Treatment with CUR+Al chloride resulted in significant increase the number of TUNEL-positive cells when compared with Altreated rats(P=0.01, Figes.4, 5). Moreover, the number of apoptotic cells were significantly decreased in rats treated with CUR alone (P=0.0001) as compared to those treated with Al alone and CUR+Al groups, but this value was not significantly different compared to control group (P>0.05).

**Fig.4:**
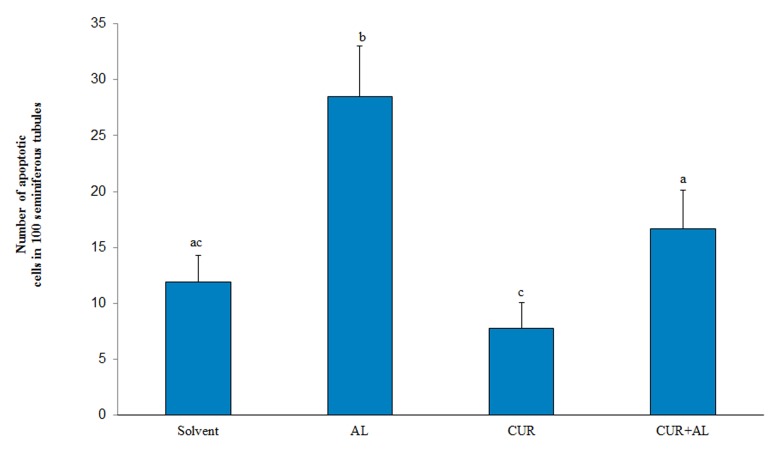
Photomicrograph of the different groups showing the number of apoptotic cells in 100 seminiferous tubules following TUNEL staining. Values are expressed as mean ± SE (n=8). Values bearing different superscript on the bar diagram vary significantly (P<0.05) using one way ANOVA and Tukey’s test. Al; Aluminum and CUR; Curcumin.

**Fig.5:**
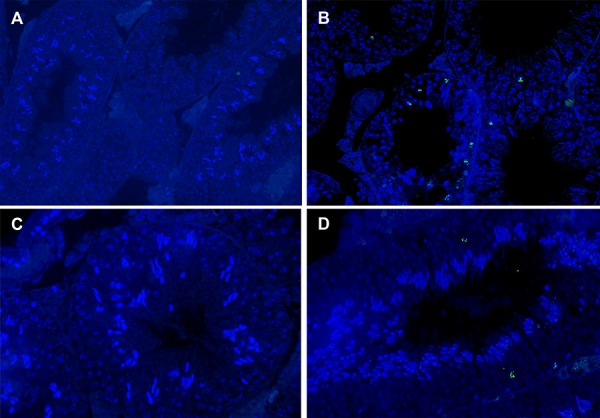
Effects of CUR and Al on the number of apoptotic cells by fluorescence microscope (magnification: ×400). A. Solvent (control), B. Treated with Al, C. Treated with CUR, and D. Treated with CUR+Al. Compared with the control group, the number of apoptotic cells were significantly increased in Al group. The apoptotic of cells (green) can be recognized. Al; Aluminum and CUR; Curcumin.

## Discussion

This study evaluated the toxic effects of Al chloride in male rat and showed that CUR had the capability to contrary Al toxicity. The present study confirmed reductions in body and testis weight following Al exposure. This is similar to previous research (6). However, the administration of CUR either alone or in combination with Al chloride could maintain testis weight at values close to the control group. This is also in accordance with previous studies (18, 21, 22). In accordance with previous findings (30), we observed significantly lower LH and testosterone levels in the Al-treated group (compared to the control group). On the other hand, since LH and testosterone levels were significantly higher in rats treated with CUR+Al than in those exposed to Al chloride alone, CUR could effectively improve sex hormone levels and decreases the harmful effects of Al. Comparable findings were also reported by previous research (31, 32). However, in present study, the FSH values remained unchanged in all groups. In contrary, in a study, by Al-Nahari and Al Eisa (33) they have showed that Al injection was significantly decreased the rate of FSH. This different is probably due to the differences in dose and duration of administration.

Al might induce such a reduction in testosterone levels by blocking calcium channels and hence down- regulating gonadotrophins secretion in the hypophysis (34). Al exposure can also suppress steroidogenesis by increasing testicular nitric oxide concentrations and decreasing cyclic adenosine monophosphate (cAMP) (10). Previous studies showed that Al injection in rat hypocamp was significantly decreased the rate of glutamate (35). Probably blocked voltage sensitive calcium cannels (VSCCs) in cells are responsible for gonadotropin- releasing hormone (GnRH) synthesis, affecting calcium influx in those cells, and decreased the GnRH secretion. Since FSH and LH secretion is promoted by FSH-releasing hormone (FSHRH) and LH-releasing hormone (LHRH) factors which are produced in separated zones in hypothalamus nucleoli, it is probable that Al inhibited LHRH production in hypothalamus, but did not effect on FSHRH production (35). In our study, rate of FSH was not affected, which may be due to the FSH synthesis mechanism that is different from LH and not affected by calcium ion.

Based on our findings, Al exposure decreased sperm quality. Likewise, previous studies documented reduced sperm count, motility, and viability following Al treatment (36, 37). Furthermore, the alteration in antioxidant system, a decrease in cAMP and an increases in nitric oxide production caused by Al treatment might have been responsible for the observed reductions in sperm motility and viability and increased morphological abnormality (12). LH stimulates the interstitial cells of the Leydig to secrete testosterone (34). Therefore, a reduction in LH and testosterone levels in the present study, which are critical to spermatogenesis, following Al exposure can justify the reduced sperm count in Al-treated rats. In our study, CUR treatment significantly improved morphological normality and sperm count, motility, and viability in rats receiving Al chloride. In other words, CUR could counteract the negative effects of Al in the mentioned- reproductive parameters. Comparable results were reported by Salama and El-Bahr (19), Sharma and Singh (21), Jalili et al. (22), Al-Nahari and Al Eisa (33).

In agreement with previous research (31), the results of this study showed that Al increased MDA level (well-known LPO indicator) and reduced SOD activity. SOD protects spermatozoa against spontaneous O_2_ toxicity and LPO. Several reports have suggested that AlCl3 may inhibit the activity of SOD. Since ROS have been indicated to have a role in steroidogenesis and gametogenesis (12), the mentioned effects might have been responsible for the reduced reproductive hormones and poor sperm quality seen in Al-treated rats. The reduction in sperm counts and sex organ weights following Al exposure in the present study can confirm the role of Al toxicity in increased oxidative stress and reinforce the role of ROS. Meanwhile, CUR has been shown to affect several targets in cells for its biological activity, while it reduced LPO and enhanced antioxidant levels in rats (13). CUR exhibits protective effects against oxidative damage by decreasing the levels of free radicals, through its free radical scavenging activity, particularly against oxygen radicals, which inhibit sulfhydryl (SH)-group oxidation. It inhibits nuclear factor kappa B (NF-kB) activity, cyclooxygenase-2 (COX-2), and mitogen-activated protein kinase (MAPK) expression, while it modulates release of several cytokines and testicular enzyme activities, mRNA expression of 17 β-hydroxysteroid dehydrogenase (17β-HSD) and cytochrome P450 sidechain cleavage (CYP450scc) enzyme in steroidogenesis (13). It seems that CUR raises testosterone and LH levels and increases the count and motility of normal sperms in treated groups through enhancing the anti-oxidant defense by increasing the expression of anti-oxidant genes in comparison with Al-treated rats.

Al cytotoxicity may be mediated by free radicals derived from this element and its capability to induce apoptosis through a wide variety of mechanisms including production of ROS, LPO, cell membrane damage, down regulation of Bax gene expression, diminished activity of alkaline phosphatase and cAMP reduction in various tissues (12, 38). In agreement with previous research (38), the results of this study showed that Al increased the amount of apoptotic cells compared with control. However, a number of studies have suggest that CUR, due to inhibition of NF-κB activation and cell scattering, can be considered as a potential therapeutic agent effective against apoptotic genes to promote cell death and proliferative processes (39). Assessment of apoptotic cells in the seminiferous tubules in the testes of rats treated with CUR+Al showed a significant reduction in the amount of apoptotic cells compared with Al group. Comparable results were reported by Aktas et al. (40). Therefore, we reported that Al induces oxidative stress and apoptosis in testicular cells, and that CUR as antioxidant prevents apoptosis induced by Al.

Histopathological analysis in the current study indicated testicular structures to be different in Al-treated rats with other groups. In fact, the Altreated group had thinner germinal epithelium and very low spermatid and sperm counts in the lumen. Similar findings have also been reported by Guo et al. (10) and Kutlubay et al. (41). This observation could be attributed to the ability of Al to cause oxidative stress, cross the blood-testis barrier, promote lipid peroxidation, and ultimately damage the biological membrane of the testis. The low sperm count, motility, and viability, as well as the high morphological abnormality, seen in Altreated rats confirm the mentioned mechanism. On the other hand, the protective effect of CUR on the testis may be demonstrated that it inhibits cellular damage and apoptosis occurring as a result of oxidative stress in the spermatogenic cells of seminiferous tubules and Leydig cells (13). Chandra et al. (42) have reported CUR to maintain normal serum testosterone levels and prevent the reduction in sex organ weights following chromium exposure. In a study on male Wistar rats, Sharma and Singh have highlighted the beneficial effects of CUR on decreasing the reproductive toxicity caused by lindane (organochlorine pesticide) (21). The protective effects of CUR have been attributed to its role in regulating LPO and boosting the antioxidant defense system. More specifically, CUR significantly decreased the levels of free radicals (through its free radical scavenging activity), induced the production of detoxification enzymes, and provided protection against degenerative diseases (43). The findings of the present study suggested that CUR treatment protected the cellular structure of the testes by increasing the formation of antioxidant products and decreasing LPO.

## Conclusion

The results of this study highlighted the protective effects of CUR on male reproductive toxicity of Al in an experimental rat model. CUR, as powerful antioxidant, was able to reduce Al-induced damage and improve sperm quality by decreasing oxidative stress. We believe that further research on the utility of CUR may indicate its usefulness as a potential treatment for spermatogenesis after testicular injury caused by Al treatment in rats.
